# Sampling scale and season influence the observed relationship between the density of deer and questing *Ixodes ricinus* nymphs

**DOI:** 10.1186/s13071-020-04369-8

**Published:** 2020-09-29

**Authors:** Eleanor R Dickinson, Caroline Millins, Roman Biek

**Affiliations:** 1 Institute of Biodiversity, Animal Health and Comparative Medicine, Graham Kerr Building, University of Glasgow, 82 Hillhead St, Glasgow, G12 8QQ Scotland, UK; 2 Scottish Centre for Ecology and the Natural Environment, Rowardennan, Glasgow, G63 0AW Scotland, UK; 3 Present Address: School of Biological Sciences, Queens University Belfast, 19 Chlorine Gardens, Belfast, BT9 5DL UK; 4 Department of Livestock and One Health, Institute of Infection, Veterinary and Ecological Sciences, University of Liverpool, Brownlow Hill, Liverpool, L69 7TX UK

**Keywords:** Tick, Parasite host relationship, Spatial distribution, Deer density, Fallow deer, Distance sampling

## Abstract

**Background:**

The relationship between environmentally transmitted tick parasites, *Ixodes* spp., and their main reproductive host, deer, is generally thought to be positive. However, measuring host abundance and density directly can be challenging and indirect methods are often used. The observed relationship between the parasite and host may be affected by sampling scale and season, which could lead to different inferences being made. Here, we aimed to test the effect of sampling scale and season on the relationship between density of deer and the density of questing *Ixodes ricinus* nymphs.

**Methods:**

The density of deer (primarily *Dama dama*) was estimated using line transect distance sampling of deer dung quantified in different seasons (winter and summer) and measured at three different nested scales (site, transect and observation level). Questing nymph density was measured using blanket drag methods and estimates were calculated at the same scales as deer density estimates. General linear models were used to evaluate the relationship between questing nymphs, deer density and other environmental variables at each sampling scale and each season deer density was measured at.

**Results:**

While a positive relationship between deer density and questing nymph density was detected at the site and transect scale, no relationship was apparent at the observation level. This was likely due to increased variation and reduced precision of deer dung counts at the finest sampling scale. Seasonal changes in deer populations were observed likely reflecting seasonal shifts in habitat usage. The summer estimates of deer density explained questing nymph density whereas winter estimates did not.

**Conclusions:**

Our results show that the scale of sampling can affect the detectability of the positive association between host and vector species. Furthermore, such associations can be obscured if hosts exhibit seasonal changes in habitat use. Thus, both sampling scale and season are important to consider when investigating the relationship between host and vector species.
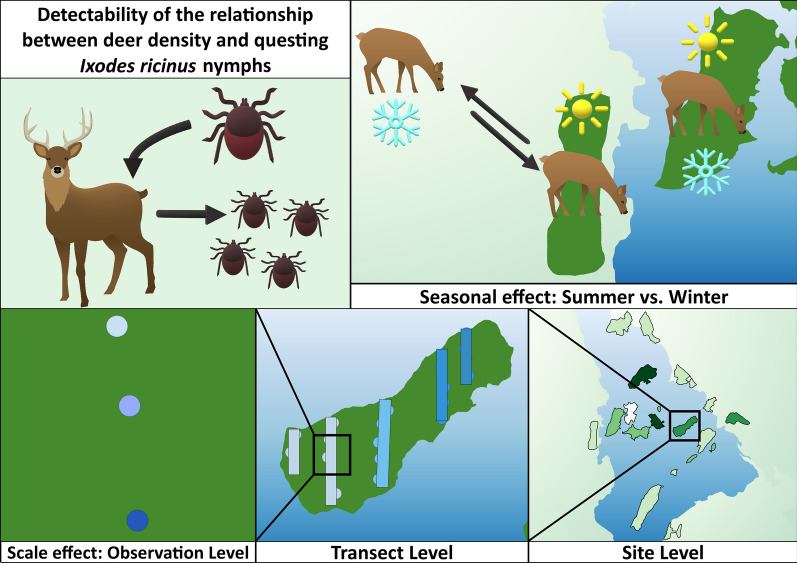

## Background

The distribution and abundance of parasites is expected to be highly dependent on host abundance, density and space use [1, 2]. In the Northern hemisphere *Ixodes ricinus* are vectors for multiple pathogens important for animal and human health such as *Borrelia burgdorferi* (*sensu lato*), the tick-borne encephalitis virus complex, *Babesia* spp. and *Anaplasma phagocytophilum* [3, 4]. The relationship between *I. ricinus* and their primary reproductive hosts, deer, is generally thought to be positive [5]. However, complexities due to multiple tick-host species and life stages of the vector may affect the presence or detectability of the relationship [6]. Furthermore, the challenge of collecting direct observational data on hosts means that information on host density and space use is rarely available and may need to be inferred from indirect observations [7].

*Ixodes ricinus* have three active life stages (larva, nymph and adult). Nymphs are considered epidemiologically most important for transmission of the emerging zoonotic pathogen *B. burgdorferi* (*s.l.*) [8, 9]. Understanding factors associated with increased nymph abundance is helpful to quantify disease risk and manage tick populations [10, 11]. Deer, as large mammals, are able to host a large number of adult ticks thereby providing opportunities for reproduction and expanding the size of the tick population [5, 12, 13]. However, there is no direct relationship between the reproductive success of adult ticks and the abundance of questing nymphs [14]. The abundance of nymphs is a result of both adult reproductive success and factors associated with larval survival. The main factors affecting larval survival are the availability of tick hosts to provide blood meals and environmental conditions which influence off-host survival [9, 11]. A range of host species including small mammals and ground foraging birds play an important role providing blood meals for larvae [15, 16]. Therefore, these other hosts and environmental factors can also influence the presence and strength of the relationship between the density of reproduction hosts (e.g. deer) and questing nymph density.

Previous studies investigating the relationship between deer and questing ticks, including nymphs, have used varying methods and scales to assess the role of deer (see Table 1). They either examined the effect of deer presence or absence (e.g. exclusion or culling [22, 26]) or measured deer density, using either direct (e.g. aerial surveys and census counts [32]) or indirect methods (e.g. surveys of deer signs or dung pellet counts [11, 33]). Sampling scale varied from local (1 m^2^–1 km^2^) to regional (1 km^2^–100 km^2^) with the relationship, if detected, varying in strength [13, 20, 22]. Generally, the effect of sampling scale is unclear. Measuring deer density at a large scale will show the overall distribution of deer over a large area. However, the spatial distribution of deer within that area will not be captured [36] and variation of other factors, such as different host species [37, 38] or even clustering of tick infestations [39], may influence whether the relationship is detectable at the smaller scale. Both from a theoretical and applied perspective, it is useful to understand at what scale the association between deer and ticks is most distinct.

**Table 1:** A comparison of studies that have investigated the relationship between ticks and deer including the scale they investigated (regional, 1 km^2^–100 km^2^; local, 1 m^2^–1 km^2^), whether the deer presence/absence was recorded through controlling deer or deer density was measured and the methods used to measure deer presence or density

Study	Deer population	Spatial scale	Methods to measure deer density	Range of deer density recorded	Observed relationship with ticks
Wilson et al. [17]	Presence/absence	Regional (single site)	–	–	Positive
Daniels et al. [18]	Presence/absence	Regional	Deer signs	–	Positive
Rand et al. [19]	Presence/absence	Regional (two sites)	–	–	Positive
Perkins et al. [20]	Presence/absence	Local	–	–	Non-linear (effect of site size)
Ruiz-Fons & Gilbert [21]	Presence/Absence	Local	Dung pellet counts	0–0.45 (deer dung index)	Positive
Gilbert et al. [22]	Presence/absence: reduced density	Multiple (regional and local)	Dung pellet counts	8–50 deer/km^2^; 0–0.25 (deer dung index)	Positive
Hofmeester et al. [5]	Presence/absence: observed deer density	Regional	Camera trapping	0.001–0.84 (camera passage rate)	Positive (presence): None (abundance)
Deblinger et al. [23]	Reduced density	Regional	Hunting records; census surveys	39–156 deer	Positive
Stafford et al. [24]	Reduced density	Regional	Marked population	97.3–13.1 deer/km^2^	Positive
Jordan et al. [25]	Reduced density	Regional	Aerial surveys	24.3–45.6 deer/km^2^	None
Kilpatrick et al. [26]	Reduced density	Regional	Aerial surveys; marked population	0–9.8 deer/km^2^	Positive
Wilson et al. [27]	Observed deer density	Regional	Dung pellet counts; deer signs	0–145 pellet groups	Positive (larvae): None (nymphs)
Wilson et al. [28]	Observed deer density	Local	Radio telemetry	0–15 deer	Positive
Rand et al. [13]	Observed deer density	Multiple (regional and local)	Hunting records; dung pellet counts	44–67 deer/km^2^	Positive
Millins et al. [11]	Observed deer density	Local	Dung pellet counts	0–0.45 (deer dung index)	None
Jordan & Schulze [29]	Observed deer density	Regional	Browsing counts	26.9–52.8 % (browse plots)	None
Ostfeld et al. [30]	Observed deer density	Regional	Hunting and browsing counts	Not reported	Positive
Gilbert et al. [31]	Observed deer density	Regional	Dung pellet counts	0.05–0.5	Positive
Tagliapietra et al. [32]	Observed deer density	Regional	Census surveys	0–24 deer/km^2^	Positive
Cagnacci et al. [33]	Observed deer density	Regional	Dung pellet counts	2–15.5 deer/100 ha	Non-linear
James et al. [10]	Observed deer density	Regional	Dung pellet counts	Not reported	Positive
Qviller et al. [34]	Observed deer density	Regional	Previous deer home range data	32–42 deer	Positive
Werden et al. [35]	Observed deer density	Regional	Dung pellet counts	0–220 pellet groups/ha	Positive

The season at which deer density was estimated may also affect the observed relationship with tick density, as deer movement and habitat use may vary seasonally [34, 40]. Measuring deer density during spring and summer coincides with the tick questing period [41], but deer density sampled during winter may provide more accurate estimates due to lower vegetation density and easier detectability of dung piles [42]. However, if deer movement and habitat use changes seasonally, deer density measured in the winter may not be relevant to nymph density, even if winter estimates are more accurate. This is because deer will only encounter questing ticks in the summer and, if deer migrate seasonally, they will only provide blood meals and affect tick populations in their summer habitat. Therefore, to test for an association between deer density and nymph density, the sampling season for estimating deer density must also be taken into account.

Using a naturally fragmented landscape with discrete habitat patches we aimed to quantify the relationship between the density of deer (primarily fallow deer, *Dama dama*) and questing nymph density of *I. ricinus* across three hierarchical spatial scales: (i) observation level as the finest scale; (ii) transect level as a medium scale; and (iii) site level as the broadest scale. Secondly, using estimates of deer density measured in different seasons (winter and summer), we aimed to test the effect of deer sampling season on the observed relationship between deer density and questing nymph density.

## Methods

### Study area

This study was conducted in Loch Lomond and the Trossachs National Park, Scotland, UK (56° 5′ N, 4° 36′ W). Twelve islands on Loch Lomond and seven mainland sites around Loch Lomond were used for this study (study area = 120 km^2^; see Additional file 1: Figure S1). The sites were predominantly woodland and between 0.03–1.15 km^2^ in area, for further information see Millins et al. [43]. Fallow deer (*D. dama*) are known to commonly occur in the study area, whereas roe deer (*Capreolus capreolus*) and sika deer (*Cervus nippon*) were observed only once in a 2008 survey and are therefore expected to be rare in this area or present at low densities [43, 44]. Red deer (*Cervus elaphus*) are not present on the islands and have not been observed in the lowland mainland sites [43]. We therefore expected fallow deer to be the dominant deer species in the study area, although other species may be present at lower densities. Small mammal and bird communities on the islands are similar to those of surrounding woodlands [43, 45]. Livestock are present in areas surrounding mainland sites, and on one island site (sheep on Inchtavannach, TA, pers. obs.).

At each site, line transects were placed in north-south orientation. The western-most transect was placed randomly within the first 200 m of the site, and subsequent transects were placed at 200 m intervals ensuring even coverage of all sites (total transect length of 26.6 km). Twenty observation points were marked along the transects at equal intervals. The different scales investigated were: (i) observation level with estimates recorded at each sampling point; (ii) transect level with estimates calculated or averaged for each transect within each site; and (iii) site level with estimates calculated or averaged for each site.

### Density of questing nymphs

The abundance of questing nymphs was estimated by collecting ticks during the peak questing period (May-July) in 2016. The blanket drag method provides an index of relative abundance of questing nymphs in the environment [10, 21]. Twenty 10-m^2^ blanket drags were conducted for each site corresponding to the observation points. Sampling was conducted once per site over a period of one to three days. A 1-m^2^ blanket was dragged across the vegetation for 10 m, within 5 m parallel to the line transect to avoid previously disturbed vegetation. Tick nymphs collected on the blanket were then counted and stored in 70% ethanol. Sampling was carried out when the vegetation was dry and between 9:00 and 16:00 h.

At the beginning and end of each drag, ground temperature and humidity were recorded (Hygro-Thermometer, ETI, Worthing, UK). Vegetation height, density and type were recorded at 3 intervals along the 10 m transect using a sward stick placed vertically in the vegetation and averaged [31]. Woodland type was determined as either mature oak and birch woodland (deciduous) or managed coniferous plantation (coniferous) [43].

### Estimated density of deer

To estimate the density of deer, two line transect surveys of deer dung using distance sampling were conducted in January-March (winter) and May-July (summer) 2016, using a previously established methodology [42]. A single observer walked each line transect which was marked at each 50 m interval using the GPS position (eTrex 10, Garmin, Olathe, Kansas, USA) and biodegradable flagging tape. Each observation of deer dung and the distance to the transect from the centre of the pellet group was recorded. Deer dung was identified to species level (see Additional file 2: Table S1), all analyses used the total deer dung observations. The dung was marked with biodegradable tape to prevent double counts of the same observation at subsequent surveys. During the second survey, vegetation type, height and density were measured, as described above, to account for the effect of vegetation growth on detection probability of dung during the summer.

At the observation level, deer density could not be estimated, we therefore used the number of deer dung observations along the transect within 100 m^2^ of each blanket drag. The density of deer was estimated at both the transect level and the site level using Distance software (version 7.3) [46], using a defecation rate of 21.4 pellet groups per deer per day, as reported for fallow deer [47], and an estimated decay rate measured for this study [48] (see Additional file 3: Table S2, Figure S2). Deer density was estimated for both the winter and summer and the results from both surveys were combined to create an average deer density (see Additional file 2: Table S1). For site level, seasonal variation (winter, summer) and vegetation density (high and low) in the summer were accounted for by stratifying data and using different probability of detection functions to estimate deer density at each site. At the transect level, data were not sufficient to stratify by vegetation density so were combined to estimate average deer density on each transect.

### Statistical analyses

All analyses were carried out using R software version 3.6.2 [49] and the *lme4* package [50]. Three separate general linear models were used to test the effect of sampling scale on the observed relationship between nymph density and estimated deer density for each level: (i) observation; (ii) transect; and (iii) site.

For the observation level model, a generalized linear mixed effect model (GLMM) was used with log-transformed number of nymphs as the response variable. Deer density measured as the number of deer dung observations was included as a fixed effect and a nested random effect of site and transect was included.

For the transect level model, a GLMM was used with log transformed mean number of nymphs per 10 m^2^ as the response variable and site as a random effect. Estimated deer density calculated for each transect was included as a fixed effect.

For the site level model, a general linear model (GLM) was used with mean number of nymphs per 10 m^2^ as the response variable. Estimated deer density calculated for each site was included as a fixed effect. All three models also included temperature, humidity, vegetation type, vegetation height and density, woodland type, proximity to the mainland and location of site (island or mainland) as fixed effects. Two-way interaction terms between vegetation height and density, and temperature and humidity were included in each model.

To investigate the effect of host estimates from different seasons, a GLM was used with the mean number of questing nymphs per 10 m^2^ as the response variable, and estimated deer density in winter and summer as main effects. To investigate the effect of sampling season on deer density estimates, a GLM was used with estimated deer density as the response variable. Location of site (island or mainland) and season (winter or summer) and their interaction were included.

All models were simplified in a step-down approach, dropping the least significant term and comparing nested models using log likelihood ratio tests (LRT). All model residuals were checked for normality. To account for zeroes in the log-transformation of the response variable, a positive constant was added.

## Results

**Fig. 1:**
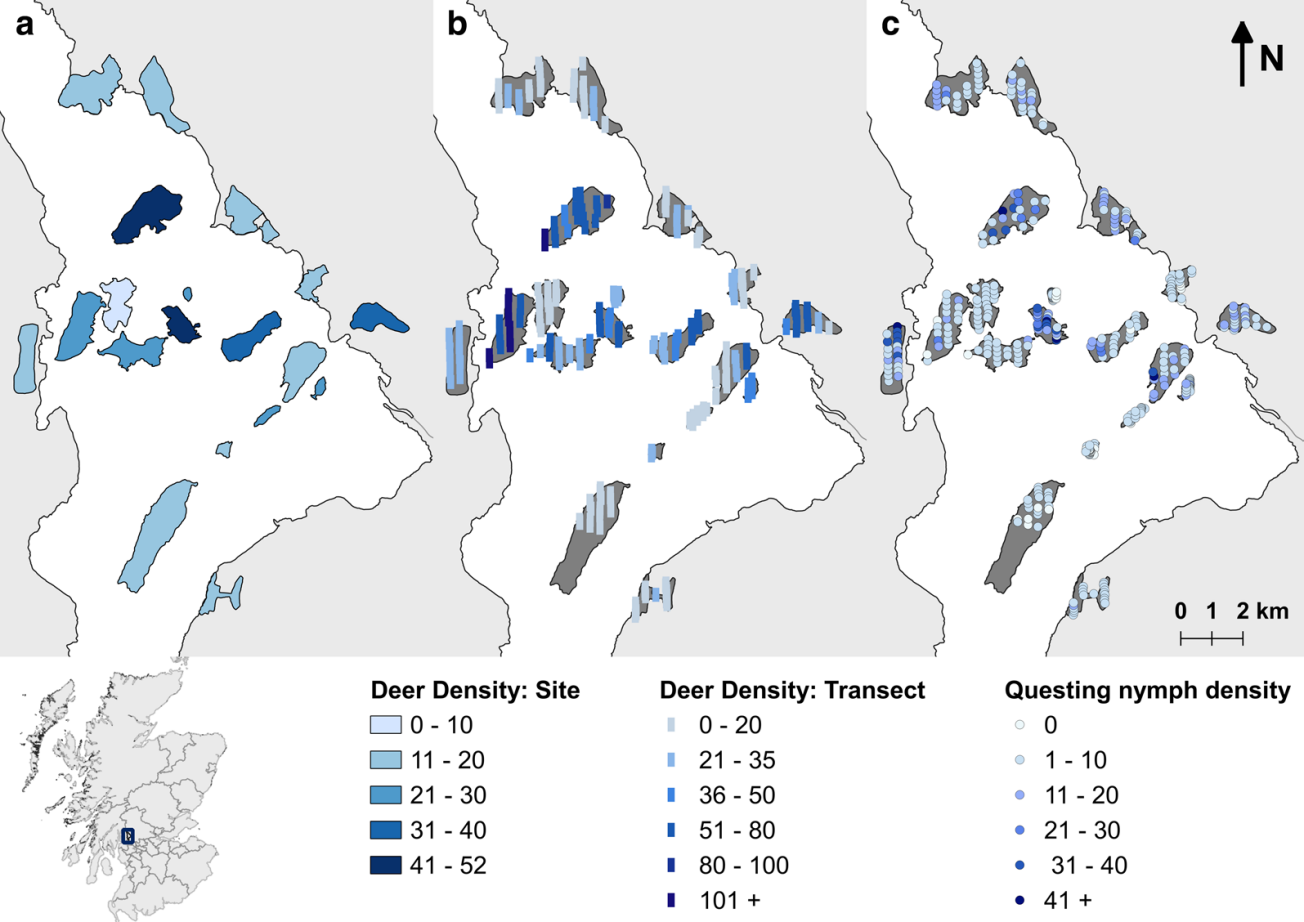
Maps of the study area showing each site shaded to the measured density of deer at site level (**a**), deer density (per km^2^) at transect level (**b**), and questing nymph density (per 10 m^2^) at each sampling location (**c**). Inset map shows the location of the study area in Scotland, UK. Made in QGIS 2.14.20 [62]

The mean number of questing nymphs measured was 0.79 nymphs per m^2^, ranging from 0.05 to 2.55 per m^2^. There was no difference between location of sites (mainland or island; Fig. 1c). Estimates were within the same range as elsewhere in Scotland (e.g. 0.1–1.6 nymphs per m^2^ [11]). The mean estimated deer density measured in the study area was 21.9 deer per km^2^, ranging from 3.1 to 53.4 deer per km^2^ (Fig. 1a; see Additional file 4: Table S3), similar to previous estimates of deer on the islands (24.6 deer per km^2^ [43]). Only 5% of the observed dung was identified as being from roe or sika deer, the remaining 95% were attributed to fallow deer (*n* = 2790/2962, see Additional file 2: Table S1). This supports our expectation that the fallow deer were the dominant deer species in the system.

### Scale effect

**Fig. 2:**
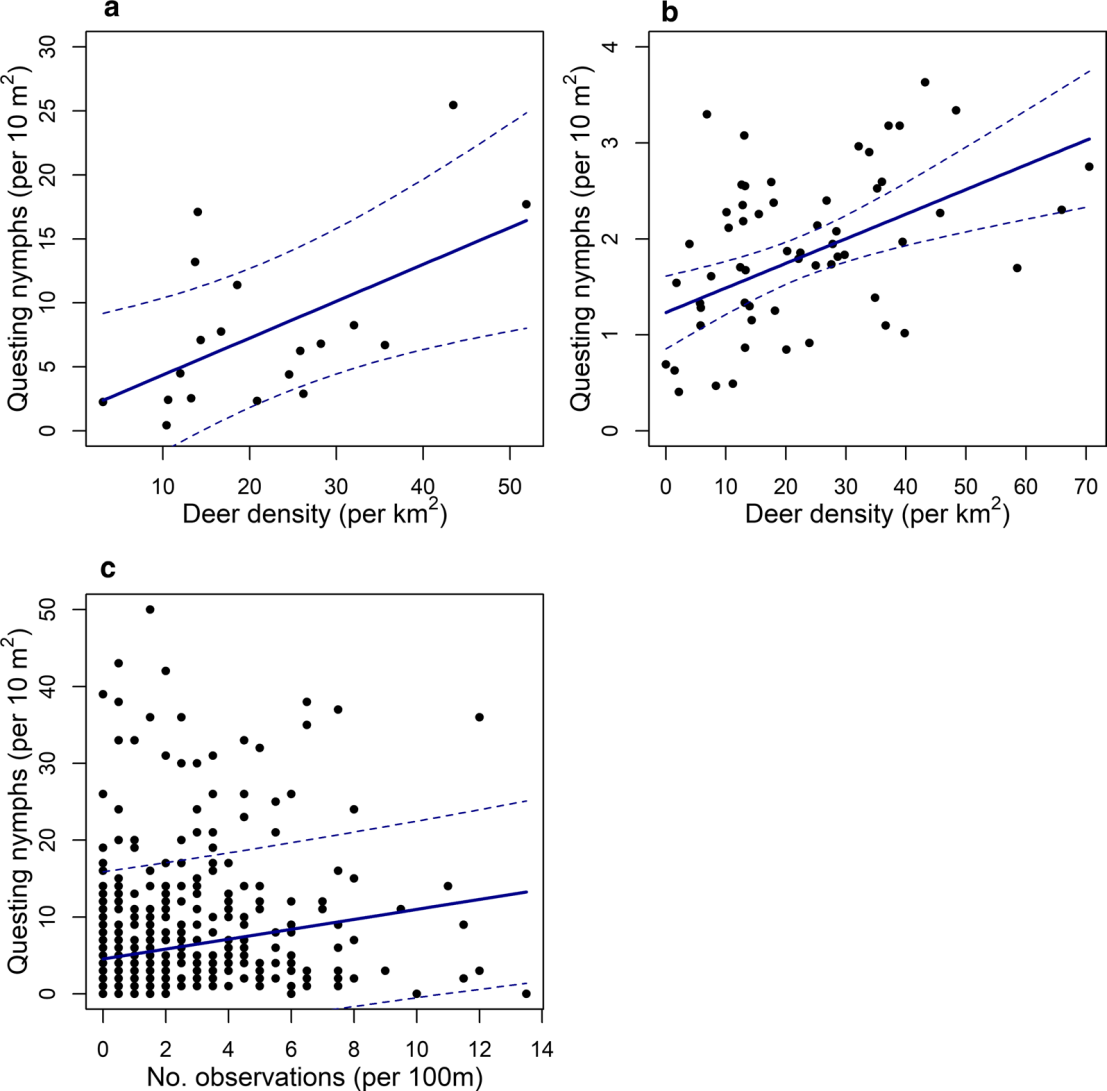
The predicted relationship (solid line) between log-transformed density of questing nymphs and estimated density of deer, at the three different sampling scales: observation (**a**), transect (**b**), site with 95% confidence intervals (dashed lines) (**c**)

At the observation level, the mean number of dung observations was 2.1, ranging from 0 to 13.5 observations. At this scale, the number of deer dung observations was not a significant predictor of the number of questing nymphs (LRT: *χ*^2^ = 0.645, *df* = 1, *P* = 0.422; Fig. 2c). The density of questing nymphs however was negatively associated with temperature (LRT: *χ*^2^ = 10.46, *df* = 1, *P* = 0.001). This model explained 57.2% of the variance of which 2.8% was explained by the fixed effect of temperature ($R^{ 2}_{\text{c}}$= 0.572, $R^{ 2}_{\text{m}}$ = 0.028), the rest of which was accounted for by the nested random effect.

At the transect level, the mean estimated density of deer was 22.35 deer per km^2^, ranging from 0 to 70.54 deer per km^2^ (Fig. 1b; see Additional file 5: Table S4). At this scale, deer density was the only significant predictor of questing nymph density (LRT: *χ*^2^ = 7.84, *df* = 1, *P* = 0.005) with a positive predicted increase of 0.26 questing nymphs per increase in deer per km^2^ (Fig 2b). This model explained 74.2% of the variance with 9.8% explained by the fixed effect, deer density ($R^{ 2}_{\text{c}}$ = 0.742, $R^{ 2}_{\text{m}}$ = 0.098).

At the site level, a positive association between nymph and deer density was detected, with a predicted increase of 0.29 questing nymphs per increase in deer per km^2^ (LRT: *χ*^2^ = 152.09, *df* = 1, *P* < 0.02; Fig. 2a). The interaction between temperature and humidity (LRT: *χ*^2^ = 119.7, *df* = 1, *P* < 0.01) was significant with nymph density predicted to increase with higher temperatures and lower humidity. Height and density of vegetation (LRT: *χ*^2^ = − 1.12, *df* = 2, *P* = 0.049) also had a marginal effect, predicting tick density to be higher at lower vegetation density and height. The model fixed effects explained 82.8% of the variance (*R*^2^ = 0.83; Table 2).

**Table 2:** Output of the models describing the relationship between nymph density and environmental determinants including deer density, each investigating the data at a different scale: (i) observation level; (ii) transect level; and (iii) site level

	Estimate	SE	*P*-value	*R*^2^
Observation level				
Intercept	2.5	0.3	< 0.001	0.57
Temperature	− 0.04	0.01	< 0.001	
Transect level				
Intercept	2.2	0.2	< 0.001	0.74
Deer density	0.01	0.008	0.003	
Site level				
Intercept	20.5	5.0	0.003	0.83
Deer density	0.02	0.01	0.04	
Temperature: humidity	0.02	0.005	0.005	
Vegetation heightM: densityL	1.0	0.9	0.3	
Vegetation heightM: densityM	− 0.5	0.8	0.6	

*Abbreviation*: SE, standard error

### Season effect

**Fig. 3:**
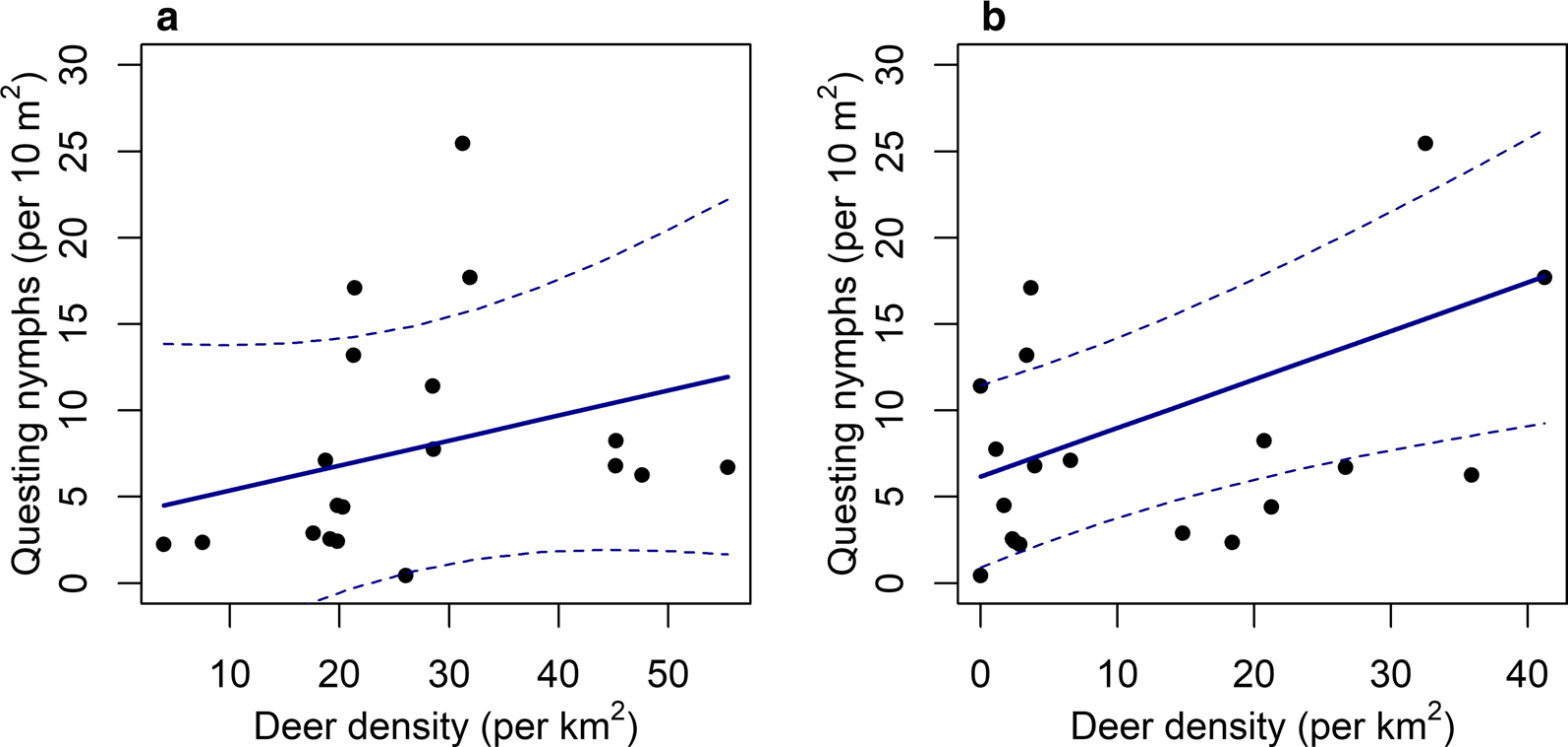
The predicted relationship (solid line) between the mean number of questing nymphs (per 10m^2^) at each site and estimated deer density (per km^2^) measured during winter (**a**) and summer (**b**), with 95% confidence intervals (dashed lines)

The winter survey estimates of deer density were not a significant predictor of questing nymph density (LRT: *χ*^2^ = 6.65, *df* = 1, *P* = 0.522; Fig. 3a). In contrast, the summer survey estimates were a strong positive predictor of questing nymph density (LRT: *χ*^2^ = 128.7, *df* = 1, *P* < 0.005; Fig. 3b; Table 3).

**Table 3:** Output of the models describing the relationship between nymph density and environmental determinants including deer density, each using estimated deer density from each season (winter and summer)

	Estimate	SE	*P*-value	*R*^2^
Winter deer estimate				
Intercept	178.1	58.3	0.01	0.66
Deer density	0.2	0.1	0.3	
Temperature: humidity	0.2	0.06	0.01	
Vegetation height M: density L	9.5	10.7	0.05	
Vegetation height M: density M	− 8.5	9.3	0.4	
Summer deer estimate				
Intercept	170.7	42.4	0.003	0.82
Deer density	0.3	0.08	0.009	
Temperature: humidity	0.2	0.04	0.005	
Vegetation height M: density L	16.6	8.1	0.01	
Vegetation height M: density M	− 2.7	7.1	0.7	

*Abbreviation*: SE, standard error

**Fig. 4:**
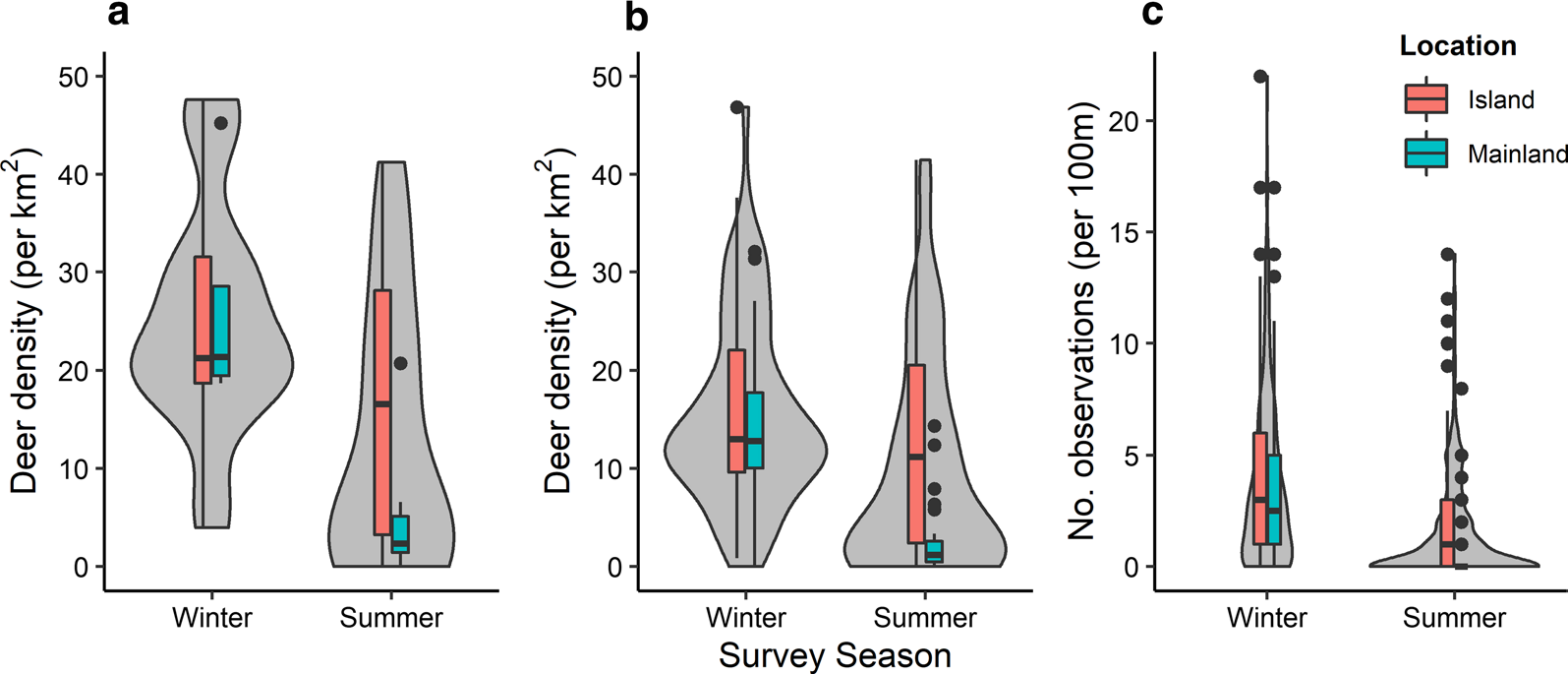
Estimated density of deer (per km^2^) measured from both surveys (winter and summer) on the mainland and island sites at different scales: site level (**a**), transect level (**b**) and observation level (the number of deer dung piles per 100 m) (**c**)

Estimates of deer density at site level were lower during the summer compared to the winter (Additional file 4: Table S3; LRT: *χ*^2^ = 9.71, *df* = 1, *P* = 0.0012), location of site (island or mainland) was not significant (LRT: *χ*^2^ = 385.6, *df* = 1, *P* = 0.14; Fig. 4a). The winter survey estimated a higher deer density at site level (mean = 27.29 deer per km^2^, SE = ± 13.51), than the summer survey (mean = 12.91 deer per km^2^, SE = ± 13.51). This difference was also detected at the transect level estimates (LRT: *χ*^2^ = 1633.3, *df* = 1, *P* < 0.001; Fig. 4b) and observation level deer dung counts (LRT: *χ*^2^ = 761.2, *df* = 1, *P* < 0.0001; Fig. 4c).

## Discussion

When investigating the relationship between deer density and questing nymph density, studies have used varying sampling scales but the effect of sampling scale on the observed relationship is generally not considered. Furthermore, although host habitat use during summer is most relevant to seasonally active tick populations, vegetation growth can affect detection of dung used to estimate deer density and may bias estimates, thus winter estimates may be more accurate. In this study, we detected a positive relationship between fallow deer density and questing nymph density at the broadest spatial sampling scale (site level) and the middle scale (transect level), but not at the finest sampling scale used (observation level). While a positive effect between deer and tick density was detected in summer, this relationship was not detectable using winter estimates of deer density.

The finest sampling scale used counts of deer dung observations and questing nymphs on 10 m^2^ blanket drags and no relationship was observed. The majority of the variance explained was by the nested random effect suggesting that there is a high degree of variation between transects and sites which is not explained by any of the measured variables. This may explain why an association between deer and nymphs was not detected at this scale. It is also important to consider the methods used, as the counts of deer dung cover a smaller area and do not account for the probability of detection, defecation or decay rates [42]. Although more robust methods to measure deer density, such as distance sampling of deer dung, require more resources, such approaches are important to consider when planning field studies on the effect of deer density on tick populations [51]. Additionally, in cases where resources allow, additional complementary methods could be used to improve accuracy of estimates (e.g. camera trapping and dung counts [52]).

At the middle and broadest sampling scales in the current study (transect and site level), estimated deer density was a significant predictor of questing nymph density [5, 10, 26]. Deer density estimates were similar at both of these scales. However, the deer density estimates at the middle scale revealed variation in habitat usage within sites that was not visible when calculating estimates at the broadest scale. Highlighting the potential usefulness of using a smaller scale to understand spatial variation in deer density [53]. Identifying areas of higher deer use, which may lead to increased tick abundance, could be applied to target vector control strategies to specific areas [54]. Habitat usage of deer may vary on a local scale for a number of reasons such as availability of resources, anti-predator behaviour and reproductive behaviour [55, 56]. Therefore, understanding and accounting for this potential variation is important for measuring deer density.

When investigating the relationship between deer and nymphs, it may also be important to consider the species of deer present [44, 57]. Deer species may differ in habitat use, affecting encounters with ticks [21], or may differ in their role to host different tick life stages [58, 59]. Although it is not known whether different deer species may affect tick populations differently, they have been shown to vary in their association with tick-borne pathogens [60]. In the present study, fallow deer were the main deer species present. Roe and sika deer, if present, are expected to be at low densities in the study area [43] and therefore considered to make a minimal contribution as tick hosts ticks in this system. However, deer population structures may vary and, if they affect tick populations differently, may need to be considered separately in their role as a primary reproduction host.

Ticks are seasonally active, and the summer estimates of deer density made during the tick questing period (May-October [9]) significantly predicted the density of questing nymphs, whereas winter estimates did not. Seasonal differences in estimated deer density were observed between winter and summer which may be explained by seasonal movement of deer [40, 56]. Winter may be more favourable for accurately estimating deer density [42] but was less relevant for explaining questing nymph density because deer movement occurs seasonally. Encounter rate of the host and vector is reduced due to low or no tick activity in areas of deer use during the winter. It is therefore important to consider deer seasonal ranges, timing of deer movement and how this may interact with tick activity [34]. Furthermore, these patterns may shift with predicted climate change as the timing of tick activity and deer movement may shift due to changes in environmental conditions [31, 58].

Patterns of habitat use in fallow deer have been shown to be variable depending on habitat availability and season supporting these conclusions [56, 61]. We observed lower deer density estimates on mainland sites during summer, but only a small seasonal effect was observed on the islands. Vegetation height was accounted for in the analyses, but growth of vegetation during the summer may have affected the detectability of dung [42], explaining reduced observations of dung in the summer. Therefore, although summer vegetation may have had an effect, a seasonal change in deer space use is consistent with our findings [56].

In addition to understanding the spatial and seasonal effect, it is also important to consider longer temporal effects. The characteristics of the *I. ricinus* life-cycle means that a stronger relationship between deer and nymphs may be observed if a time lag is taken into consideration. As deer are important for feeding adult ticks, the length of time between successful adult feeding on deer and questing nymph activity may be important to quantify [9]. If deer density and space use are consistent over time, this time lag may not have a significant effect. However, as deer density is likely to vary with factors such as resource availability [56], there may be further complexity in the association between deer density and questing nymph density. If deer density changes, either naturally or by human intervention, the effect on ticks is more likely to be seen in larvae before it is observed in nymphs [23, 27]. Measuring how the relationship between deer density and larval and nymph density changes over time may improve understanding of the effect of these mechanisms.

## Conclusions

This study provides empirical evidence that spatial scale and season affects the detectability and strength of the relationship between deer density and nymph density. While a positive relationship was found at two broader sampling scales, it was not detected at the finer spatial scale. The intermediate scale used in this study (i.e. transect level) detected within-site variation not measured at the broadest scale (i.e. site level). This study also highlights that winter estimates of deer density were not a useful predictor of questing nymph abundance, while summer estimates were. To optimise study design, it is also important to consider the effect of seasonal changes in host and vector distribution. Improving understanding of tick population drivers, including the role of different deer species and quantifying the time lag between host abundance and density of different tick life stages, will facilitate vector management strategies in a changing climate.

## Supplementary information

**Additional file 1: Figure S1.** A map showing the study area of Loch Lomond, which is located in the south-west of Scotland as seen in the insert map. Dark grey areas denote each of the 12 island sites and 7 mainland sites used in the study, with their corresponding labels. The map was created in ArcGIS.

**Additional file 2: Table S1.** Models estimating the density of fallow deer, with different key functions and series expansion terms, were tested [1]. The model outputs for the estimated deer density at the two scales (site and transect) and for summer, winter, and combined estimates. Site level estimates were post-stratified by each site, and transect level estimates were post-stratified by each transect at each site. Survey effort and the number of observations for each survey is reported. Estimated deer dung decay rates (winter = 85.60 ± 3.46 days, summer = 80.37 ± 3.13 days, combined = 80.20 ± 4.47 days) and defecation rate of 21.4 pellet groups per deer per day [2], were used. Fallow deer were used based on knowledge of the deer in the area [3] and identification of the dung [4]. Roe deer and Sika deer were recorded once in the study area in 2008 (Jimmy Irvine, Scottish Natural Heritage personal communication [3]), 5.8% of dung was visually identified as not being fallow deer (*n* = 172/2962). Defecation rates for these species are similar [1, 5], therefore would not lead to bias in estimated density if included. No observations of red deer dung were recorded. The Akaike’s information criterion (AIC) was compared and the model with the lowest difference (∆AIC) was selected. ƒ(0) is the probability detection function of the perpendicular distances. Pooled estimates of the density of individuals (D) in the study area are shown, upper and lower 95% confidence limits (LCL and UCL) and the percentage coefficient of variation (%CV).

**Additional file 3: Table S2.** To measure the length of time to dung decay, a representative sample of fresh dung pellets were marked in December 2015 at the beginning of the first survey (*n* = 119), and for the second survey fresh dung pellets were marked in March 2016 prior to the survey (*n* = 658). At the end of both surveys, these sites were returned to and the age and proportion of surviving pellets that could be relocated was recorded. The mean time to decay was estimated using the proportion of pellets surviving the period of time from the beginning of the respective survey to the end which was then modelled as a function of age using logistic regression model as outlined by Laing et al. [1]. Decay rates were first calculated independently for each survey, as well as calculating overall decay rate by fitting a single model combining all data. **Figure S2.** Logistic regression predicting the time for dung to decay with both surveys combined.

**Additional file 4: Table S3.** Estimated deer density (deer per km^2^) at each site during the winter and summer surveys, and the combined estimate of deer density, with estimated percentage coefficient of variation (%CV).

**Additional file 5: Table S4.** Estimated deer density (deer per km^2^) at each transect at each site during the winter and summer surveys, and the combined estimate of deer density, with estimated percentage coefficient of variation (%CV).

## Data Availability

Data supporting the conclusions of this article are included within the article. The datasets used and/or analysed during the present study are available from the corresponding author on reasonable request.

## Abbreviations

GLMM: Generalized liner mixed effects model
GLM: General linear model
LRT: Likelihood ratio test
SE: Standard error
