# Supplementary figures and images for: Sampling scale and season influence the observed relationship between the density of deer and questing Ixodes ricinus nymphs
Source: Parasit Vectors. 2020 Sep 29;13:493. doi: 10.1186/s13071-020-04369-8 (PMC7526098; doi:10.1186/s13071-020-04369-8)

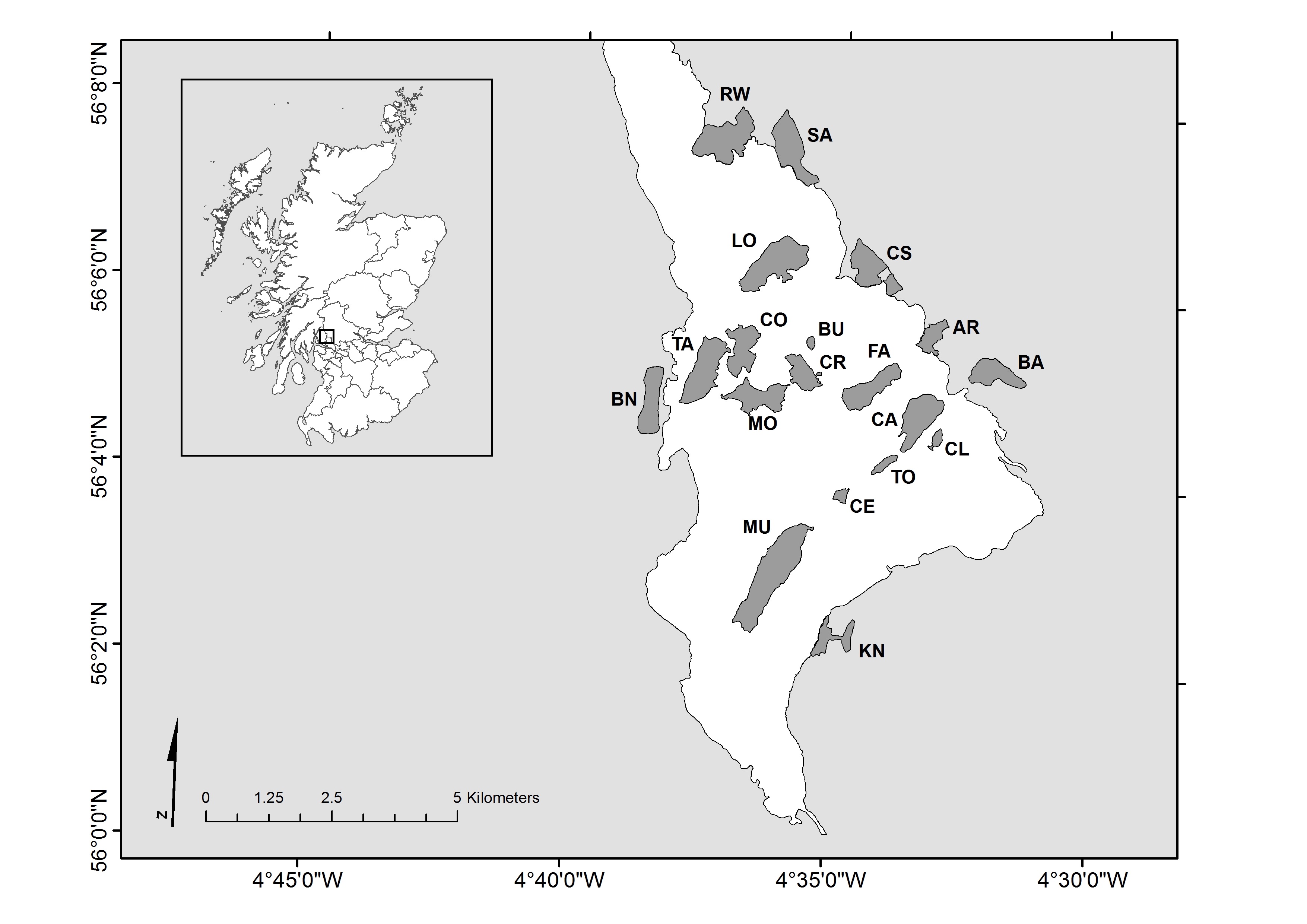

Supplement: Supplementary file 1 — Additional file 1: Figure S1. A map showing the study area of Loch Lomond, which is located in the south-west of Scotland as seen in the insert map. Dark grey areas denote each of the 12 island sites and 7 mainland sites used in the study, with their corresponding labels. The map was created in ArcGIS. [file 13071_2020_4369_MOESM1_ESM.jpg]
