# Supplementary material for: Sampling scale and season influence the observed relationship between the density of deer and questing Ixodes ricinus nymphs
Source: Parasit Vectors. 2020 Sep 29;13:493. doi: 10.1186/s13071-020-04369-8 (PMC7526098; doi:10.1186/s13071-020-04369-8)
Supplement: Supplementary file 2 — Additional file 2: Table S1. Models estimating the density of fallow deer, with different key functions and series expansion terms, were tested [1]. The model outputs for the estimated deer density at the two scales (site and transect) and for summer, winter, and combined estimates. Site level estimates were post-stratified by each site, and transect level estimates were post-stratified by each transect at each site. Survey effort and the number of observations for each survey is reported. Estimated deer dung decay rates (winter = 85.60 ± 3.46 days, summer = 80.37 ± 3.13 days, combined = 80.20 ± 4.47 days) and defecation rate of 21.4 pellet groups per deer per day [2], were used. Fallow deer were used based on knowledge of the deer in the area [3] and identification of the dung [4]. Roe deer and Sika deer were recorded once in the study area in 2008 (Jimmy Irvine, Scottish Natural Heritage personal communication [3]), 5.8% of dung was visually identified as not being fallow deer (n = 172/2962). Defecation rates for these species are similar [1, 5], therefore would not lead to bias in estimated density if included. No observations of red deer dung were recorded. The Akaike’s information criterion (AIC) was compared and the model with the lowest difference (∆AIC) was selected. ƒ(0) is the probability detection function of the perpendicular distances. Pooled estimates of the density of individuals (D) in the study area are shown, upper and lower 95% confidence limits (LCL and UCL) and the percentage coefficient of variation (%CV). [file 13071_2020_4369_MOESM2_ESM.pdf]

Additional file 2: Table S1: Models estimating the density of fallow deer, with different key functions and series expansion terms, were tested [1]. The model outputs for the estimated deer density at the two scales (site and transect) and for summer, winter and combined estimates. Site level estimates were post-stratified by each site, and transect level estimates were post stratified by each transect at each site. Survey effort and the number of observations for each survey is reported. Estimated deer dung decay rates (winter = 85.60 ± 3.46 days, summer = 80.37 ± 3.13 days, combined = 80.20 ± 4.47 days) and defecation rate of 21.4 pellet groups per deer per day [2], were used. Fallow deer were used based on knowledge of the deer in the area [3] and identification of the dung [4]. Roe deer and sika deer were recorded once in the study area in 2008 (Jimmy Irvine, Scottish Natural Heritage personal communication [3]), 5.8% of dung was visually identified as not being fallow deer (n= 172/2962). Defecation rates for these species are similar [1,5], therefore would not lead to bias in estimated density if included. No observations of red deer dung were recorded. The Akaike's information criterion (AIC) was compared and the model with the lowest difference (ΔAIC) was selected. $f(0)$ is the probability detection function of the perpendicular distances. Pooled estimates of the density of individuals (D) in the study area are shown, upper and lower 95% confidence limits (LCL and UCL) and the percentage coefficient of variation (%CV).

Scale	Survey	Effort (m)	No. observations	Model and adjustment terms	ΔAIC	$f(0)$	Deer density (deer per km^2)	LCL	UCL	%CV
**Site**	Winter	31417	2167	Hazard-rate polynomial	0	0.64	27.29	22.73	32.76	0.090
				Uniform cosine	2.0	0.62	28.23	23.63	33.74	0.087
				Half – normal cosine	17.6	0.63	27.58	23.06	32.98	0.088
				Half-normal hermite polynomial	17.6	0.63	27.58	23.06	32.98	0.088
	Summer	25362	768	Hazard-rate polynomial	0	0.55	12.91	10.64	15.67	0.095
				Half – normal cosine	2.7	0.57	12.65	10.22	15.65	0.107
				Half-normal hermite polynomial	4.2	0.57	12.50	10.10	15.47	0.107
				Uniform cosine	6.4	0.53	13.63	11.36	16.35	0.088
	Combined	56779	2935	Hazard-rate polynomial	0	0.62	21.87	17.83	26.44	0.099
				Half – normal cosine	11.7	0.59	22.61	18.62	27.45	0.098
				Half-normal hermite polynomial	27.3	0.60	22.37	18.41	27.18	0.098
				Uniform cosine	27.3	0.60	22.37	18.41	27.18	0.098
**Transect**	Winter	26604	2167	Hazard-rate polynomial	0	0.64	35.12	33.30	37.04	0.027
				Half – normal cosine	2.0	0.62	36.33	35.25	37.45	0.015
				Half-normal hermite polynomial	17.6	0.63	35.48	34.24	36.77	0.018
				Uniform cosine	17.6	0.63	35.48	34.24	36.77	0.018
	Summer	24455	768	Hazard-rate polynomial	0	0.55	16.62	15.40	17.95	0.039
				Half – normal cosine	2.7	0.57	16.28	14.42	18.38	0.062
				Half-normal hermite polynomial	4.2	0.57	16.09	14.24	18.18	0.062
				Uniform cosine	6.4	0.53	17.54	17.03	18.06	0.015
	Combined	51059	2935	Hazard-rate polynomial	0	0.62	22.35	18.29	27.74	0.081
				Uniform cosine	11.7	0.59	24.01	19.86	28.91	0.079
				Half-normal cosine	27.3	0.60	23.72	19.60	28.60	0.079
				Half-normal hermite polynomial	27.3	0.60	23.72	19.60	28.60	0.079

References

1. Marques FFC, Buckland ST, Goffin D, Dixon CE, Borchers DL, Mayle BA, et al. Estimating deer abundance from line transect surveys of dung: Sika deer in southern Scotland. J Appl Ecol. 2001;38:349–63.
2. Mayle B, Peace A, Gill R. How Many Deer? A guide to estimating deer population size. Forestry Commission field book. Edinburgh: Forestry Commission. 1999;18:96.Accessed 12 December 2015
3. Millins C, Dickinson ER, Isakovic P, Gilbert L, Wojciechowska A, Paterson V, et al. Landscape structure affects the prevalence and distribution of a tick-borne zoonotic pathogen. Parasites and Vectors. 2018;11:1–11.
4. Howie A. 2016. An identification guide to the Deer of Cornwall and the Isles of Scilly, http://northpennines.wp-sites.durham.gov.uk/wp-content/uploads/sites/37/2016/11/deer_ID_guide.pdf
5. Mitchell B, Rowe JJ, Ratcliffe P, Hinge M. Defecation frequency in Roe deer (*Capreolus capreolus*) in relation to the accumulation rates of faecal deposits. J Zool London. 1985;1–7.
