# Supplementary material for: Sampling scale and season influence the observed relationship between the density of deer and questing Ixodes ricinus nymphs
Source: Parasit Vectors. 2020 Sep 29;13:493. doi: 10.1186/s13071-020-04369-8 (PMC7526098; doi:10.1186/s13071-020-04369-8)
Supplement: Supplementary file 3 — Additional file 3: Table S2. To measure the length of time to dung decay, a representative sample of fresh dung pellets were marked in December 2015 at the beginning of the first survey (n = 119), and for the second survey fresh dung pellets were marked in March 2016 prior to the survey (n = 658). At the end of both surveys, these sites were returned to and the age and proportion of surviving pellets that could be relocated was recorded. The mean time to decay was estimated using the proportion of pellets surviving the period of time from the beginning of the respective survey to the end which was then modelled as a function of age using logistic regression model as outlined by Laing et al. [1]. Decay rates were first calculated independently for each survey, as well as calculating overall decay rate by fitting a single model combining all data. Figure S2. Logistic regression predicting the time for dung to decay with both surveys combined. [file 13071_2020_4369_MOESM3_ESM.pdf]

Additional file 3: Table S2: To measure the length of time to dung decay, a representative sample of fresh dung pellets were marked in December 2015 at the beginning of the first survey (n=119), and for the second survey fresh dung pellets were marked in March 2016 prior to the survey (n=658). At the end of both surveys, these sites were returned to and the age and proportion of surviving pellets that could be relocated was recorded. The mean time to decay was estimated using the proportion of pellets surviving the period of time from the beginning of the respective survey to the end which was then modelled as a function of age using logistic regression model as outlined by Laing et al. [1]. Decay rates were first calculated independently for each survey, as well as calculating overall decay rate by fitting a single model combining all data.

Survey	Number dung observations	Number of dung persisting	Age	Decay rate	SE
Winter	119	119	0	85.60	3.46
		76	91		
		40	100		
Summer	658	658	0	80.37	3.13
		550	98		
		507	104		
		144	115		
		63	120		
Combined	777	777	0	80.20	4.47
		734	91		
		626	98		
		590	100		
		547	104		
		184	115		
		103	120		

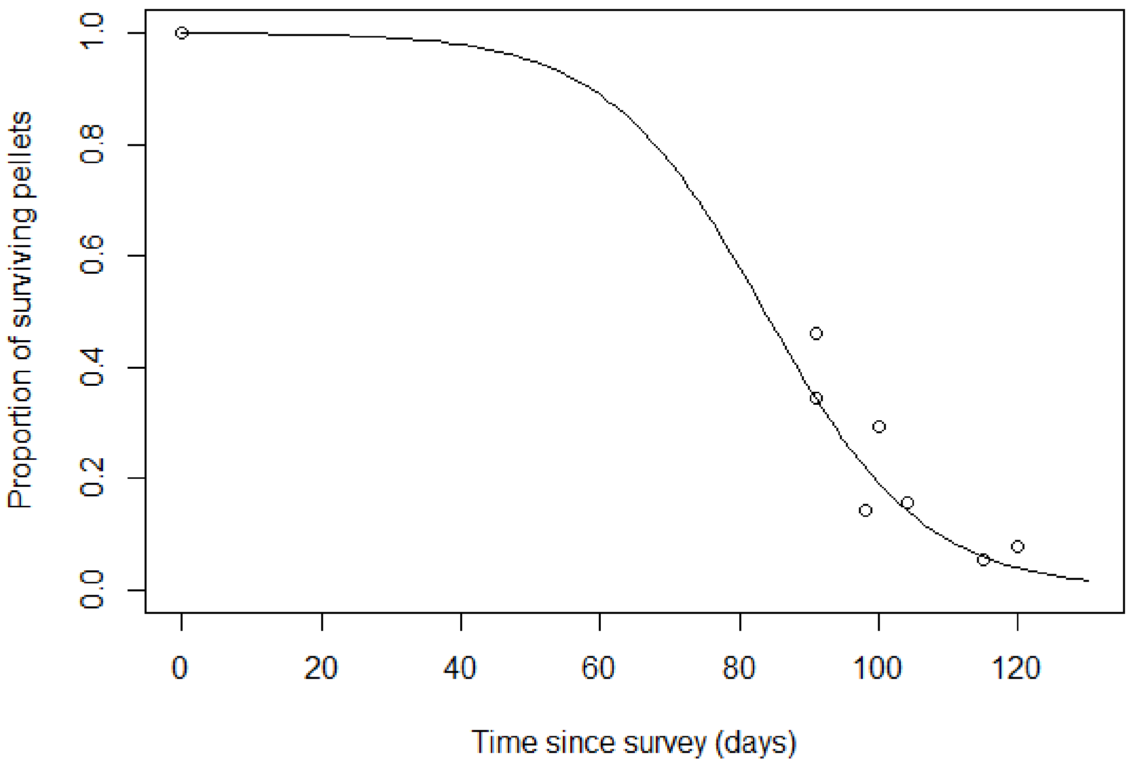

Figure S2: Logistic regression predicting the time for dung to decay with both surveys combined.

References

1. Laing SE, Buckland ST, Burns RW, Lambie D, Amphlett A. Dung and nest surveys: estimating decay rates. J Appl Ecol. 2003;40:1102–11.
