# Supplementary material for: Sampling scale and season influence the observed relationship between the density of deer and questing Ixodes ricinus nymphs
Source: Parasit Vectors. 2020 Sep 29;13:493. doi: 10.1186/s13071-020-04369-8 (PMC7526098; doi:10.1186/s13071-020-04369-8)
Supplement: Supplementary file 4 — Additional file 4: Table S3. Estimated deer density (deer per km2) at each site during the winter and summer surveys, and the combined estimate of deer density, with estimated percentage coefficient of variation (%CV). [file 13071_2020_4369_MOESM4_ESM.pdf]

Additional file 4: Table S3: Estimated deer density (deer per km^2) at each site during the winter and summer surveys, and the combined estimate of deer density, with estimated percentage coefficient of variation (CV).

Location	Site	Area (km^2)	Winter survey		Summer survey		Combined Estimate	
			Estimate	CV	Estimate	CV	Estimate	CV
Island	BU	0.031	7.5	2.8	18.4	3.9	20.9	3.3
Island	CA	0.53	21.3	28.7	3.4	73.3	13.7	51.0
Island	CE	0.057	26.0	2.8	0	0	10.4	1.4
Island	CL	0.056	45.2	2.8	4.0	3.9	28.2	3.3
Island	CO	0.42	4.0	36.7	2.9	50.1	3.1	43.4
Island	CR	0.28	31.2	10.8	32.5	18.0	43.5	14.4
Island	FA	0.45	55.4	21.6	26.7	16.9	53.4	19.3
Island	LO	0.75	31.9	18.6	41.2	16.4	51.9	17.5
Island	MO	0.45	20.3	17.1	21.3	32.5	24.6	24.8
Island	MU	1.15	19.8	24.4	2.5	46.8	10.6	35.6
Island	TA	0.63	47.6	20.6	35.9	19.7	48.7	20.2
Island	TO	0.075	17.6	11.3	14.8	16.3	26.2	13.8
Mainland	AR	0.2	19.1	17.0	2.3	100.1	13.3	58.5
Mainland	BA	0.45	45.2	21.5	20.7	18.1	32.0	19.8
Mainland	BN	0.55	21.4	2.9	3.7	76.5	14.0	39.7
Mainland	CS	0.49	28.5	13.2	0	0	18.6	6.6
Mainland	KN	0.54	19.8	37.1	1.7	21.0	12.0	29.0
Mainland	RW	0.74	28.7	26.3	1.1	97.4	16.7	61.9
Mainland	SA	0.63	18.7	38.0	6.6	42.7	14.4	40.3
