# Supplementary material for: Sampling scale and season influence the observed relationship between the density of deer and questing Ixodes ricinus nymphs
Source: Parasit Vectors. 2020 Sep 29;13:493. doi: 10.1186/s13071-020-04369-8 (PMC7526098; doi:10.1186/s13071-020-04369-8)
Supplement: Supplementary file 5 — Additional file 5: Table S4. Estimated deer density (deer per km2) at each transect at each site during the winter and summer surveys, and the combined estimate of deer density, with estimated percentage coefficient of variation (%CV). [file 13071_2020_4369_MOESM5_ESM.pdf]

Additional file 5: Table S4: Estimated deer density (deer per km^2) at each transect at each site during the winter and summer surveys, and the combined estimate of deer density, with estimated percentage coefficient of variation (%CV).

Location	Site	Transect	Transect length (m)	Estimated deer density (deer per km^2)			%CV
				Winter	Summer	Combined	
Island	BU	T1	371	11.5	28.3	22.9	23.0
Island	CA	T1	413	5.9	0.9	12.3	29.0
		T2	857	9.0	1.4	18.8	16.4
		T3	367	15.5	2.5	32.4	19.0
		T4	314	24.0	3.8	50.0	16.6
Island	CE	T1	115	10.4	0	29.5	37.9
Island	CL	T1	265	26.3	2.3	48.1	18.4
Island	CO	T1	680	3.4	2.4	11.2	23.7
		T2	750	0.9	0.6	2.8	44.8
		T3	400	1.3	0.9	4.3	50.1
Island	CR	T1	347	19.1	19.9	62.4	14.2
		T2	450	23.7	24.7	40.3	11.9
		T3	350	18.2	18.9	60.7	14.5
Island	FA	T1	450	23.8	11.4	31.4	14.6
		T2	500	24.3	11.7	32.1	12.2
		T3	474	31.4	15.1	41.4	10.8
		T4	350	46.9	22.6	61.8	10.2
		T5	300	57.0	27.4	75.9	10.1
Island	LO	T1	234	32.1	41.5	136.1	11.8
		T2	500	30.8	39.8	66.9	9.3
		T3	450	18.8	24.3	43.5	11.4
		T4	600	14.0	18.1	61.6	11.1
		T5	650	14.8	19.1	63.4	10.4
		T6	600	28.4	36.7	60.7	8.3
		T7	200	20.5	26.5	87.1	15.8
Island	MO	T1	21	12.0	12.6	40.4	70.7
		T2	126	9.0	9.5	30.3	33.4
		T3	416	17.9	18.8	61.3	13.2
		T4	415	12.2	12.8	21.1	16.6
		T5	250	6.1	6.4	20.4	29.0
		T6	490	6.4	6.8	21.7	20.1
		T7	200	13.9	14.6	48.8	21.4
Island	MU	T1	400	9.4	1.2	12.2	26.8
		T2	100	5.2	0.7	6.8	57.8
		T3	300	11.7	1.5	15.3	23.7
		T4	250	7.4	0.9	9.7	27.8
Island	TA	T1	240	4.6	3.4	143.3	11.4
		T2	634	37.6	28.4	56.4	8.1
		T3	1000	33.4	25.2	100.6	6.9
		T4	600	17.0	12.8	51.0	12.0
Island	TO	T1	200	13.0	10.9	16.4	25.9
		T2	140	9.9	8.3	12.5	35.4
Mainland	AR	T1	450	12.4	1.5	23.6	20.1
		T2	450	10.0	1.2	18.9	22.5
		T3	150	17.9	2.2	17.0	40.9
Mainland	BA	T1	100	17.3	7.9	42.5	31.7
		T2	514	31.4	14.4	77.7	10.6
		T3	378	27.0	12.4	68.5	13.2
		T4	100	13.9	6.4	34.0	35.4
		T5	100	5.2	2.4	12.7	57.8

Mainland	BN	T1	950	13.2	2.3	26.3	13.2
		T2	1050	11.2	1.9	22.2	13.7
Mainland	CS	T1	500	17.6	0	17.5	16.6
		T2	600	26.8	0	26.6	14.7
		T3	100	12.8	0	12.7	57.8
		T4	300	10.1	0	10.0	27.8
Mainland	KN	T1	285	1.7	0.1	1.5	100.0
		T2	300	20.4	1.8	18.3	21.9
		T3	200	32.1	2.8	28.8	25.1
		T4	412	5.2	0.5	4.7	33.4
Mainland	RW	T1	400	12.7	0.5	12.5	20.5
		T2	392	12.1	0.5	23.8	21.4
		T3	323	26.5	1.1	26.1	14.7
		T4	257	12.8	0.5	12.6	25.9
		T5	686	13.8	0.6	13.5	17.5
Mainland	SA	T1	450	2.9	1.0	4.3	37.9
		T2	600	9.6	3.4	13.9	15.8
		T3	300	16.6	5.8	24.2	16.6
		T4	350	0	0	0	0
